# Longitudinal Progression of Traumatic Bone Marrow Lesions Following Anterior Cruciate Ligament Injury: Associations With Knee Pain and Concomitant Injuries

**DOI:** 10.1002/jor.70067

**Published:** 2025-09-20

**Authors:** Callie E. Stirling, Nina Pavlovic, Sarah L. Manske, Richard E. A. Walker, Steven K. Boyd

**Affiliations:** ^1^ Department of Biomedical Engineering, Schulich School of Engineering University of Calgary Calgary Canada; ^2^ McCaig Institute for Bone and Joint Health, Cumming School of Medicine University of Calgary Calgary Canada; ^3^ Department of Radiology, Cumming School of Medicine University of Calgary Calgary Canada

**Keywords:** ACL, Bone, Diagnostic Imaging, Osteoarthritis, Osteoarthritis ‐ Post Traumatic, Reconstruction

## Abstract

Traumatic bone marrow lesions (BMLs) occur in ~80% of anterior cruciate ligament (ACL) injuries, typically in the lateral femoral condyle (LFC) and lateral tibial plateau (LTP). Associated with microfractures, vascular proliferation, inflammation, and bone density changes, BMLs may contribute to posttraumatic osteoarthritis. However, their relationship with knee pain is unclear. This study examined the prevalence, characteristics, and progression of BMLs after ACL injury, focusing on associations with pain, meniscal and ligament injuries, and fractures. Participants (*N* = 100, aged 14–55) with MRI‐confirmed ACL tears were scanned within 6 weeks post‐injury (mean = 30.0, SD = 9.6 days). BML volumes were quantified using a validated machine learning method, and pain assessed via the Knee Injury and Osteoarthritis Outcome Score (KOOS). Analyses included *t*‐tests, Mann–Whitney U, chi‐square, and Spearman correlations with false discovery rate correction. BMLs were present in 95% of participants, primarily in the LFC and LTP. Males had 33% greater volumes than females (*p* < 0.05), even after adjusting for BMI. Volumes were higher in cases with depression fractures (*p* = 0.022) and negatively associated with baseline KOOS Symptoms. At 1 year, 92.68% of lesions (based on lesion counts) resolved in Nonsurgical participants, with a 96.13% volume reduction (*p* < 0.001). KOOS outcomes were similar between groups, except for slightly better Pain scores in the Nonsurgical group. Baseline Pain and Sport scores predicted follow‐up outcomes. BMLs are common post‐ACL injury, vary by sex and fracture status, and modestly relate to early symptoms. Most resolve within a year, with limited long‐term differences by surgical status.

**Abbreviations**
ACL: Anterior cruciate ligament
ACL‐R: ACL‐Reconstruction
BML: Bone marrow lesion
MRI: Magnetic resonance imaging
OA: Osteoarthritis

## 1 Introduction

Traumatic bone marrow lesions (BMLs), also known as bone bruises or bone contusions, occur in approximately 80% of anterior cruciate ligament (ACL) injuries [1, 2]. They often result from high‐force impact between the femur and tibia during injury. These lesions appear as high‐signal intensity regions on fluid‐sensitive magnetic resonance imaging (MRI) sequences, including T2‐weighted images, and as low‐signal intensity regions on T1‐weighted images. BMLs are associated with microfractures, vascular proliferation, and increased inflammation and have been identified as sites of concentrated short‐term bone density changes [2, 3].

Traumatic BMLs typically occur in the lateral compartment, notably in the lateral femoral condyle (LFC) and posterior lateral tibial plateau (LTP), regions linked to pivot‐shift or multi‐planar valgus loading mechanisms during ACL injury [4, 5]. Although research on sex‐specific BML patterns is limited, one study reported a significantly higher prevalence of LTP bruising in females, suggesting potential sex‐based differences [4]. BML characteristics early after injury may serve as indicators of injury severity, with larger lesions possibly contributing to knee pain or marking the onset of posttraumatic osteoarthritis (OA) [6, 7, 8, 9]. BML volume has been associated with a prolonged return to sport, particularly after ACL reconstruction (ACL‐R) [10]. Even after the resolution of BMLs, cartilage changes may persist, suggesting a link to long‐term joint degeneration [11]. A study of ACL‐injured individuals found no significant association between knee pain and BML volume in individual regions but suggested that fractures might obscure this relationship [9]. The study found that greater knee pain was weakly associated with larger BML volumes in the medial compartment, entire knee, and non‐depression fracture regions. A recent study found that deep osteochondral lesions extending into subchondral bone have been linked to significantly worse patient‐reported outcomes 10 years after ACL‐R [12]. Other studies have suggested correlation between BML volume and functional outcomes, but systematic reviews do not support this [13]. In idiopathic OA, however, BMLs are commonly known to be strongly associated with pain [14].

This study aims to investigate BMLs following knee ACL injury, with a focus on their relationship to knee pain and associated joint damage, leveraging longitudinal follow‐up of patient‐reported outcomes and imaging. First, we will evaluate the baseline prevalence and characteristics of BMLs within 6 weeks of injury, including their associations with meniscal tears, ligament injuries, and fractures. Second, we will examine the longitudinal progression of BMLs to determine whether they persist, resolve, or change location over time. Third, we will assess whether the presence of BMLs at baseline is associated with increased knee pain at follow‐up, using Knee Injury and Osteoarthritis Outcome Score (KOOS) data, with a particular focus on individuals who do not undergo ACL‐R. By addressing these objectives, we aim to better understand the development and clinical significance of BMLs in the context of post‐injury knee pain and recovery.

## 2 Methods

### 2.1 Datasets

**Figure 1:**
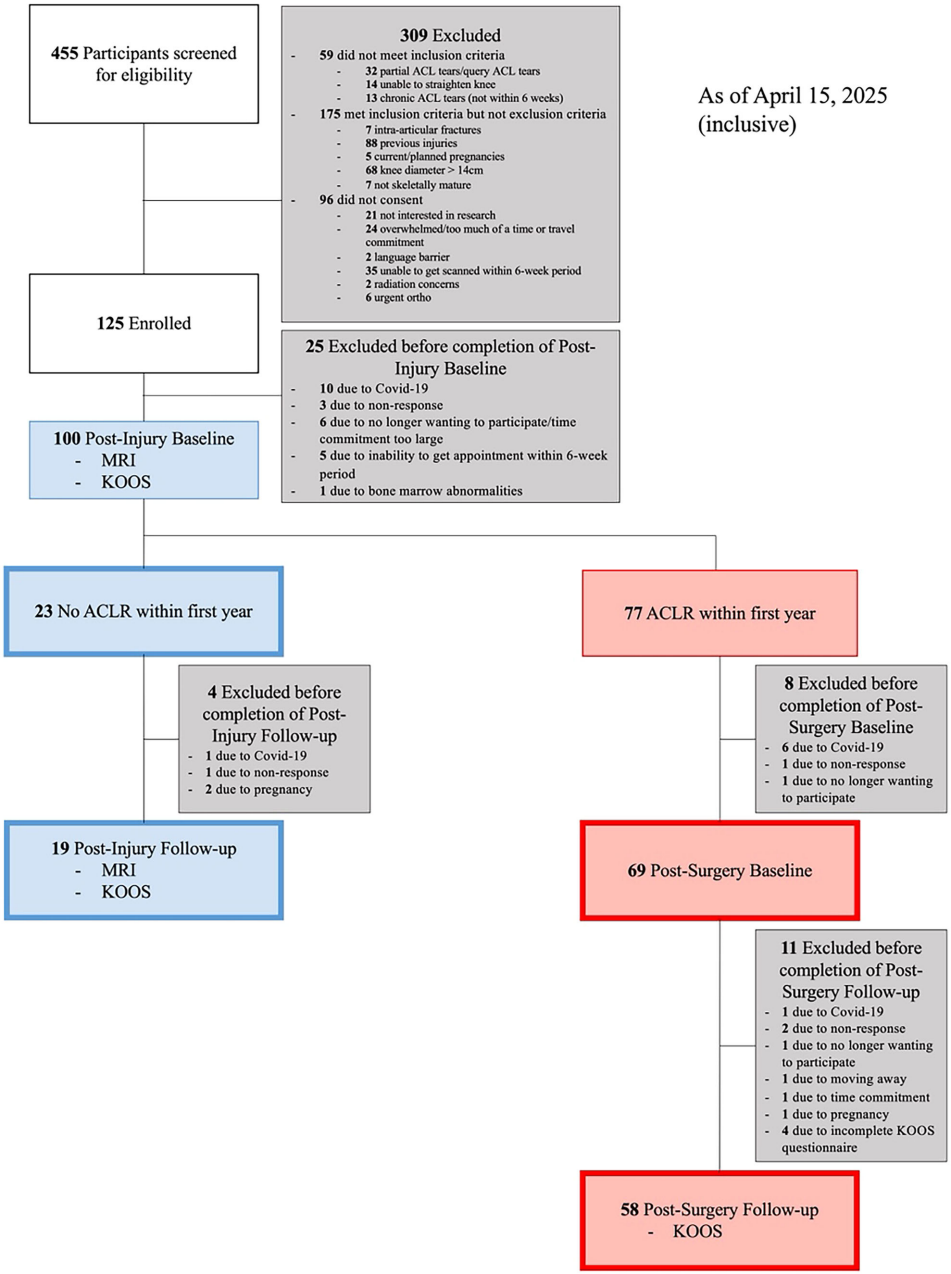
Flowchart outlining recruitment, exclusion, enrollment, imaging, and withdrawal processes for the study.

This study is part of an ongoing longitudinal prospective observational cohort study, approved by the Conjoint Health Research Ethics Board at the University of Calgary (REB19–1184). Study data was collected and managed using the Research Electronic Data Capture (REDCap) system, hosted at the University of Calgary [15]. Participants were recruited from several partner clinics following an ACL tear. The inclusion criteria for the study were individuals between 14 and 55 years of age, with clinical evidence of a unilateral, complete ACL tear. Participants were excluded if they met any of the following exclusion criteria: radiographic confirmation of intra‐articular fracture(s), prior knee ligament tear(s) in either leg, pregnancy (current or planned within the next year, recent (< 12 months) history of disease and/or treatment that affects bone turnover, radiographic evidence of skeletal immaturity, metal in scan region, or previous injuries or implants that are not MRI‐safe. Figure 1 shows the recruitment process.

Participants underwent baseline imaging approximately 6 weeks after ACL injury, followed by annual imaging thereafter. As this is an observational study, we did not influence treatment decisions; the choice to undergo ACL‐R, and its timing, is made by participants in consultation with their physician. If a participant has ACL‐R during the study, a new postsurgical baseline MRI is performed approximately 6 weeks after surgery. From that point on, follow‐up imaging is reset to align with the surgery date and continues annually.

### 2.2 Magnetic Resonance Imaging

MRI scans were obtained within approximately 6 weeks post‐injury using a 1.5‐T MR scanner (GE OptimaMR430S, 1.5 T, Waukesha, WI, USA). The imaging protocol included T2‐weighted fat‐suppressed fast spin echo images [TR/TE, 4300/56 ms; echo train length (ETL), 11; matrix, 320 × 256; field of view (FOV), 140 mm; slice thickness, 3.5 mm; gap, 0.3 mm;] for evaluating BMLs.

Each participant's injured leg was scanned at approximately 6 weeks post‐injury with sagittal, axial and coronal proton density MRI and sagittal fat‐suppressed T2‐weighted MRI. Images were reviewed prospectively by a subspecialty trained MSK radiologist to confirm a complete ACL tear and to assess concomitant meniscal injury (medial and lateral meniscus tears), additional ligament or tendon tears, chondral injuries, depression fractures, and other relevant tissue pathologies.

### 2.3 Bml Volume Calculation

All MRI scans were semantically segmented using the automated methods developed in our lab [16]. Total volumes were then calculated for each participant using custom scripts (Python, version 3.9.12). After segmentation, the number of lesions were manually counted in each compartment and bone.

### 2.4 Pain Measurement

Pain and symptoms of the injured knee were measured using KOOS [17], a validated patient‐reported outcome measure designed to evaluate both short‐ and long‐term knee‐related symptoms and function. The KOOS consists of five subscales: Symptoms, Pain, Activities of Daily Living (ADL), Sport and Recreation Function, and Knee‐related Quality of Life (QoL). Each item is scored on a 5‐point Likert scale and transformed to a 0–100 scale, with higher scores indicating better outcomes (i.e., fewer symptoms or limitations).

### 2.5 Statistical Analysis

Changes in BML volume between baseline and follow‐up were assessed using the Shapiro–Wilk test to check for normality. If differences were normally distributed, a paired *t*‐test was used to assess the changes in BML volume between baseline and follow‐up. To compare non‐parametric continuous data between independent groups, the Mann–Whitney U test was used to. Categorical variables were analyzed using Pearson's Chi‐squared test and relationships between continuous variables were evaluated using Spearman's rank correlation. To account for multiple comparisons, false discovery rate correction was applied using the Benjamini‐Hochberg procedure. Associations were considered statistically significant at a false discovery rate threshold of Q < 0.1. Values were reported with statistical significance considered at *p* < 0.05.

## 3 Results

A total of 125 eligible participants were recruited to the study, 101 of which attended the baseline study visit. The study occurred during the Covid‐19 pandemic, which made recruitment challenging. One participant was excluded due to bone marrow abnormalities observed on imaging by the subspecialty trained MSK radiologist. Mean age of participants (*N* = 100) was 33.6 (min–max 15–55, SD 10.7), composed of 68 females and 32 males. Participant demographic information and BML characteristics are summarized in Table 1.

**Table 1:** Baseline Characteristics.

Variable	Female	Male	Total	% Incidence
Participant Count	68	32	100	—
BML Volume Baseline (Mean ± SD) [cm³]	15.48 ± 12.33	22.99 ± 15.82	17.88 ± 13.92	—
BML Volume Baseline (Min) [cm³]	0	0.35	0	—
BML Volume Baseline (Max) [cm³]	48.77	74.74	74.74	—
Zero BML Volume Count	5	0	5	—
Partial ACL Tear Count	4	2	6	6.0%
Complete ACL Tear Count	64	30	94	94.0%
Medial Meniscal Tear	38	19	57	57.0%
Lateral Meniscal Tear	35	18	53	53.0%
Total Meniscal Tear	51	26	77	77.0%
All Depression Fractures	15	12	27	27.0%
Depression Fracture – MFC (Osteochondral)	0	0	0	0.0%
Depression Fracture – MTP Rim (Impaction)	3	3	6	6.0%
Depression Fracture – LFC Sulcus Terminalis	1	4	5	5.0%
Depression Fracture – LTP Posterior Margin	13	7	20	20.0%
Partial Thickness MCL Tear	44	18	62	62.0%
Low‐Grade Partial Thickness PCL Tear	7	4	11	11.0%
Partial Thickness LCL Tear	16	13	29	29.0%

After 1 year of follow‐up only 19 participants had not undergone ACL‐R since their baseline visit. Fifty‐eight participants (42 females, 16 males) underwent surgery within the first‐year post‐injury, at a mean time of 165.6 days (SD = 85.1). These participants completed a follow‐up KOOS survey at a mean of 581.5 days (SD = 84.8) after injury. The remaining participants withdrew from the study or did not complete follow‐up KOOS Surveys.

### 3.1 Baseline Injury Characteristics and Early Functional Outcomes

**Figure 2:**
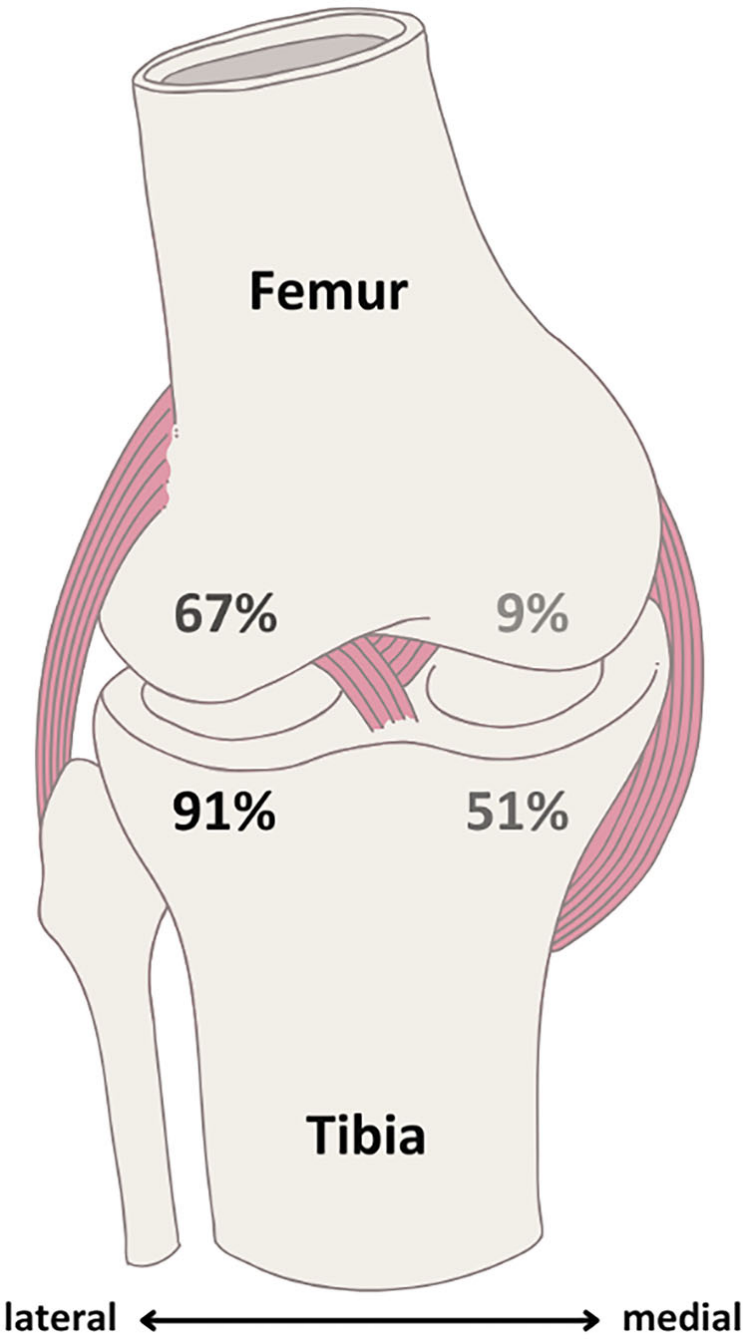
Distribution of presence of BML patterns across different regions.

ACL injuries resulting from sports were most frequently associated with skiing (*N* = 44; 35 female, 9 male), basketball (*N* = 8; 2 female, 6 male), and soccer (*N* = 7; 4 female, 3 male). The mean time from injury to baseline MRI was 30.0 (SD 9.6) days. BMLs were present in 95/100 participants with a mean total volume of 17.88 (SD 13.92) cm³ per participant. BML patterns were most commonly observed in the lateral tibial plateau (LTP) and lateral femoral condyle (LFC). The distribution of BMLs by anatomical location is illustrated in Figure 2, highlighting the predominance in the LTP and LFC.

**Figure 3:**
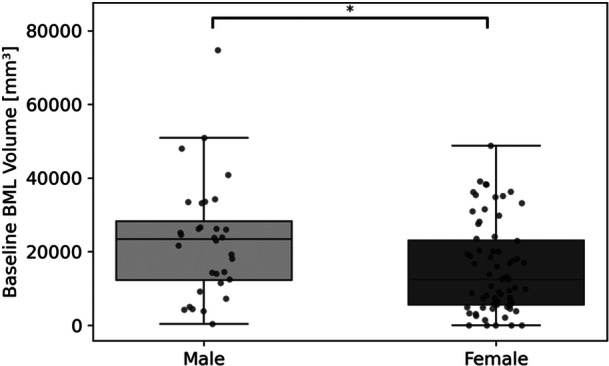
Distribution of baseline BML volumes for male and female sexes. *Indicates significant difference by Mann‐Whitney U test.

**Figure 4:**
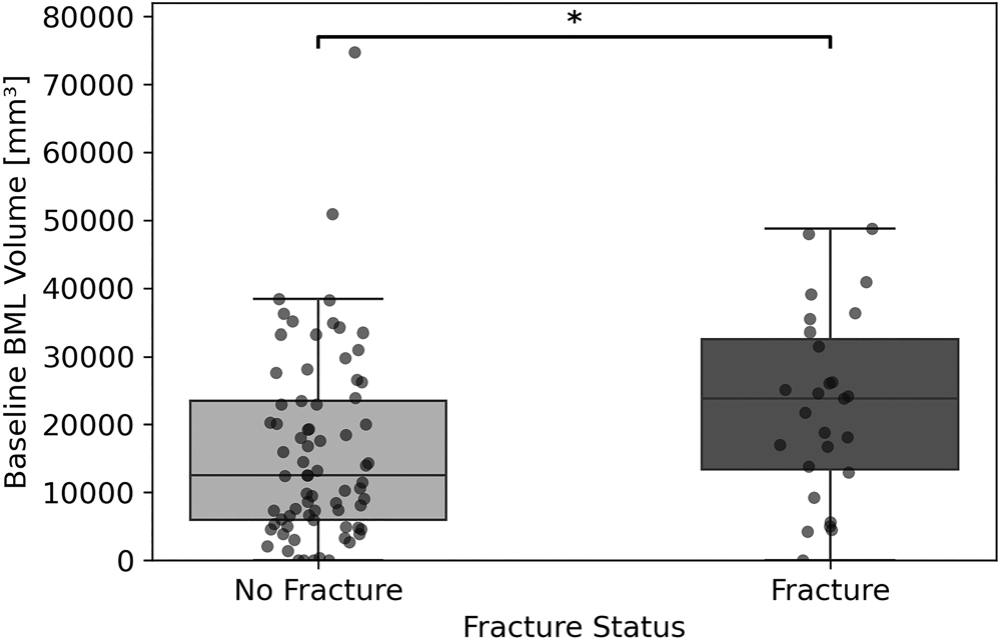
Baseline BML volume comparison between participants with depression fractures and those without fracture present. *Indicates a statistically significant difference between groups.

The Mann–Whitney U test was conducted to compare baseline BML volumes between males and females (Figure 3). The results showed a significant difference between the sexes (*U* = 1404, *p* = 0.020), with males exhibiting higher median BML volumes than females (+33% difference). To account for potential confounding by body mass index (BMI), BML volumes were adjusted, and group difference remained statistically significant (*U* = 1388, *p* = 0.027), reinforcing the observed sex‐based disparity. Notably, BMI alone was not significantly associated with BML volume, nor was the relationship between absolute BML volume and BMI. This suggests that other factors—such as joint injury or biomechanical loading—may play a more substantial role in BML development and progression. The Mann‐Whitney U test between baseline BML volumes of participants with and without depression fractures found a significant difference, with those exhibiting depression fractures demonstrating higher baseline BML volumes (*U* = 689.00, *p* = 0.022). This finding suggests that the presence of a depression fracture is associated with increased BML volume at baseline (Figure 4).

In addition to observed sex differences in BML volume, surgical status was moderately negatively correlated with age (*r* = −0.332, *p* = 0.001, *Q* = 0.013), reflecting that younger individuals were more likely to undergo surgery. Fracture status was also positively correlated with age (*r* = 0.291, *p* = 0.005, *Q* = 0.038), indicating a moderate association between risk of depression fracture and older age. The BML volume at baseline was negatively correlated with KOOS Symptoms at baseline (*r* = −0.266, *p* = 0.010, *Q* = 0.063), implying that higher BML volumes may be associated with more severe symptoms. Additionally, age was moderately negatively correlated with KOOS Sport at baseline (*r* = −0.258, *p* = 0.012, *Q* = 0.075), suggesting that older individuals may experience greater functional limitations in sports.

The KOOS subscales showed several significant positive correlations. KOOS Symptoms score at baseline was moderately correlated with both KOOS Pain (*r* = 0.500, *p* < 0.001, *Q* < 0.001) and KOOS Sport (*r* = 0.372, *p* < 0.001, *Q* = 0.003), suggesting that individuals with more severe symptoms also experience greater pain and functional limitations in sports. KOOS Pain was also moderately correlated with KOOS QoL (*r* = 0.317, *p* = 0.002, *Q* = 0.018), indicating that pain severity impacts overall quality of life. Furthermore, KOOS Pain was strongly correlated with KOOS Sport (*r* = 0.529, *p* < 0.001, *Q* < 0.001), emphasizing the role of pain in limiting sports function. Finally, KOOS QoL was positively correlated with KOOS Sport (*r* = 0.520, *p* < 0.001, *Q* < 0.001), highlighting the importance of sports participation in overall quality of life.

Analysis of injury patterns revealed significant associations between medial collateral ligament (MCL) and lateral collateral ligament (LCL) tears (χ² = 6.54, *p* = 0.011, *Q* = 0.070) and between posterior cruciate ligament (PCL) and LCL tears (χ² = 13.8, *p* < 0.001, *Q* = 0.003), suggesting that these ligament tears may share common mechanisms of injury. Further analysis revealed that co‐occurring MCL and LCL tears were associated with higher baseline BML volumes (*r* = 0.267, *p* = 0.010, *Q* = 0.049), and that patients with all three ligament tears (MCL, LCL, and PCL) also exhibited elevated BML volumes (*r* = 0.236, *p* = 0.023, *Q* = 0.094), indicating that multiple ligament injuries may contribute additively to BMLseverity. Notably, the MCL injuries observed were primarily partial‐thickness tears, while the PCL injuries were low‐grade, further indicating that even lower‐severity injuries to these stabilizing structures tend to co‐occur with LCL involvement. Among skiers, females had a higher proportion of MCL tears (26 of 35; 74.3%), and skiing was significantly associated with MCL injuries (χ² = 9.49, *p* = 0.002, *Q* = 0.078).

### 3.2 Longitudinal Observations of BML Resolution

**Figure 5:**
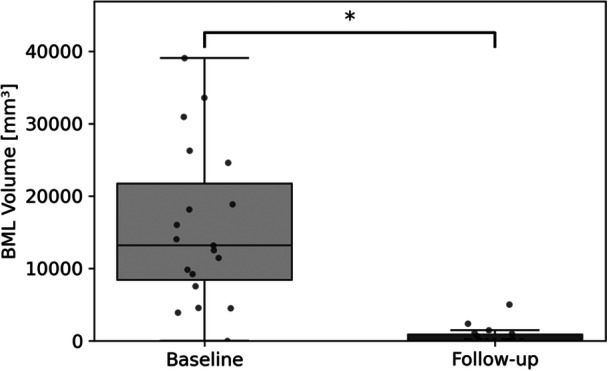
Change in total BML volume from baseline to one‐year follow‐up (*n* = 19) in Nonsurgical participants. *Indicates a statistically significant difference, indicating a notable reduction in BML volume.

After 1 year of follow‐up, 19 participants had not undergone ACL‐R since their baseline visit. The mean time from injury to the follow‐up scan was 402.3 ± 19.3 days. Among these participants, 41 BMLs were identified at baseline through manual lesion counting. At 1 year of follow‐up, 38 of these 41 BMLs (92.68%) had fully resolved. Regionally, 1 medial femoral, 12 lateral femoral, 7 medial tibial, and 16 lateral tibial lesions disappeared over time. Three lesions did not fully resolve by follow‐up: one in the one in the lateral femur, one in the medial tibia, and one in the lateral tibia, each occurring in a different participant. Although most lesions fully resolved, we also assessed total BML volume using automated segmentation to capture more subtle changes. Across participants, there was a 96.13% average reduction in total BML volume from baseline to follow‐up (*p* < 0.001), reflecting both the resolution of most lesions and shrinkage in those that persisted. This supports the visual count data while offering a quantitative confirmation of the overall decline in BML burden (Figure 5).

### 3.3 Longitudinal Knee Outcome and Association with Baseline BML Volume

**Figure 6:**
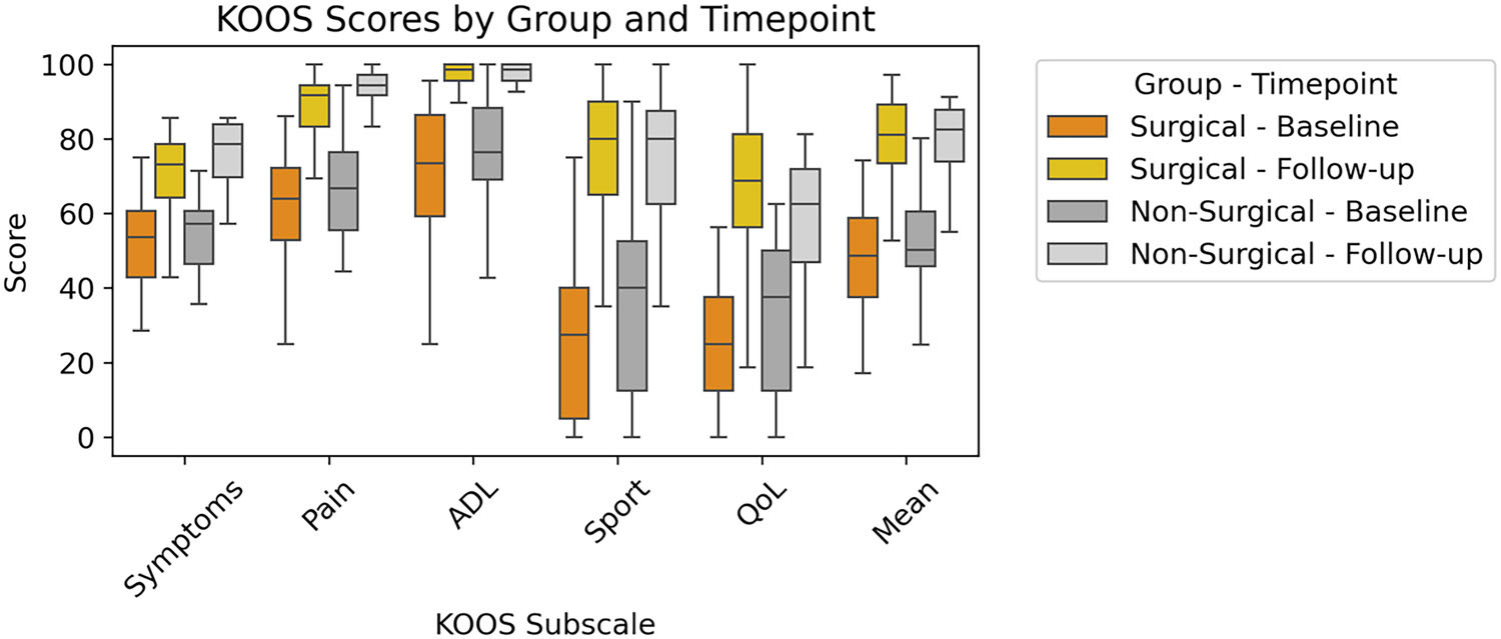
Comparison of KOOS subscale scores between surgical and Nonsurgical groups at baseline and follow‐up. Mean scores for each KOOS domain (Symptoms, Pain, Activities of Daily Living [ADL], Sport, and Quality of Life [QoL] BML) are presented for both groups. Higher KOOS scores indicate better outcomes, reflecting fewer symptoms, less pain, and better function and QoL.

A total of 58 participants (42 females, 16 males) completed both the baseline scan and the 1‐year follow‐up KOOS surveys and had surgery within the first year post‐injury. Follow‐up surveys were conducted 581.5 days (SD 84.8) days after injury. On average, surgery occurred 165.6 days (SD 85.1) days post‐injury. When pooled with Nonsurgical participants who also completed the follow‐up survey (*n* = 77; 52 females, 25 males), the mean time from injury to follow‐up was 536.0 ± 108.9 days. Surgical and Nonsurgical groups were pooled due to the lack of significant differences in KOOS outcomes at follow‐up (Figure 6, Table 2). While the follow‐up pain scores between groups was significantly different (*p* = 0.049), indicating slightly better outcomes in the Nonsurgical group, no other KOOS subscale or mean scores showed significant differences between groups. Notably, mean follow‐up scores were nearly identical (79.9 vs. 79.7; *p* = 0.920) suggesting that surgery does not appear to confer a meaningful overall benefit in patient‐reported outcomes compared to Nonsurgical management.

**Table 2:** KOOS subscale scores comparing surgical and Nonsurgical participants at baseline and follow‐up. Differences between groups at each timepoint were assessed using the Mann–Whitney U test.

	KOOS subscale	Surgical mean (*N* = 58)	Nonsurgical mean (*N* = 19)	*p*‐value
Baseline	Symptoms	52.2	54.1	0.577
	Pain	61.7	67.1	0.255
	ADL	71.5	75.3	0.338
	Sport	27.5	36.6	0.194
	QoL	24.6	31.2	0.170
	**Mean**	**47.5**	**52.9**	**0.221**
Follow‐up	Symptoms	71.4	76.1	0.103
	Pain	88.2	92.7	0.049
	ADL	95.5	96.4	0.917
	Sport	76.4	75.5	1.000
	QoL	67.9	57.9	0.105
	**Mean**	**79.9**	**79.7**	**0.920**

The KOOS subscale scores exhibited significant longitudinal associations, reinforcing the predictive relationships between baseline and follow‐up outcomes. The baseline KOOS Pain score demonstrated strong correlations with follow‐up KOOS Symptoms (*r* = 0.495, *p* < 0.001, *Q* < 0.001) and follow‐up KOOS Pain (r = 0.539, *p* < 0.001, *Q* < 0.001), indicating that initial pain levels are strongly predictive of future pain and symptom severity. Additionally, baseline KOOS Sport was moderately correlated with both follow‐up KOOS Symptoms (*r* = 0.326, *p* = 0.011, *Q* = 0.062) and follow‐up KOOS Sport (*r* = 0.321, *p* = 0.012, *Q* = 0.065), indicating that early sports function is a predictor of both future symptoms and sports outcomes during recovery.

## 4 Discussion

This study longitudinally assessed BML volume and associated knee pain following ACL injury, which aligns with prior work [9] that found no strong relationship between BML volume and knee symptoms within the first month post‐injury. We extend that work to focus on the longitudinal progression of BML volume and its potential link to pain over a longer period. Our findings show that, although baseline BML volume was not strongly correlated with knee pain during the acute phase of injury, BMLs largely resolved over time. Although baseline BML volume was modestly associated with KOOS Symptoms at baseline, this relationship was limited and did not persist at follow‐up. Importantly, we found that baseline pain and sports function scores were strong predictors of future outcomes, and surgery did not provide a meaningful overall benefit in patient‐reported outcomes compared to Nonsurgical management. This is consistent with previous research showing that the KOOS Sport and QoL subscales are among the most responsive to meaningful clinical change, supporting their use in monitoring recovery and identifying patients likely to improve [18]. Our results suggest that the resolution of BMLs over time may not be directly related to pain relief or functional improvements, and that early pain and functional status are more critical in predicting long‐term outcomes following ACL injury. BML volume did not significantly predict any other follow‐up KOOS scores. These results highlight that pain and recovery following ACL injury are likely influenced by a complex interplay of structural damage, such as ligament or cartilage injury, and nonstructural factors including inflammation, neuromuscular adaptations, and psychosocial components. However, several other associations identified in this study provide additional context for understanding symptom variability and recovery outcomes beyond BML volume alone.

Sex differences emerged as a factor in BML volume, with males having higher BML volumes than females. This difference could not be explained simply by body size, however, because BML volume was not associated with BMI. The lack of association between BML volume and BMI suggests that anatomical differences between males and females may play a role. This aligns with previous research suggesting sex‐based differences in knee morphology and injury progression [19, 20]. One potential factor is joint congruence, which differs between sexes and has been linked to knee stability; lower congruence in females may contribute to altered load distribution and BML development [21].

The high prevalence of concomitant injuries in our sample, including frequent co‐occurrence of MCL and LCL injuries, supports previous reports on the complexity of ACL trauma. MCL involvement is common, and lateral meniscal tears frequently occur with ACL injuries [22, 23]. Our study found a similar pattern, confirming that there is a frequent consequence of ACL‐MCL injuries. Notably, skiing—particularly among females—was associated with a higher proportion of MCL tears, highlighting sport‐ and sex‐specific patterns of ligament involvement that may be related to valgus‐external rotation forces encountered during skiing [24]. This study also identified significant associations between PCL and LCL injuries, a combination less commonly reported in the literature. Importantly, patients with multiple concomitant ligament injuries, including MCL, LCL, and PCL tears, tended to have higher baseline BMLvolumes, suggesting that the combination of injuries may reflect greater injury severity. These findings imply that more extensive ligament involvement could result from higher‐energy trauma mechanisms [25], contributing to larger lesion volumes and potentially more complex joint pathology. We found BMLs are more commonly present in the lateral femoral condyle and tibial plateau, patterns that are consistent with known ACL injury mechanics, which often involve pivot‐shift or valgus loading forces that stress the lateral compartment [4]. Interestingly, participants with depression fractures had significantly higher BML volumes, supporting the notion that more severe bone trauma results in larger BMLs, which was also observed by Driban and colleagues [9]. This association suggests that BMLs may serve as a useful surrogate marker for underlying microarchitectural damage in the setting of acute trauma. Notably, fracture status was positively correlated with age, which may reflect age‐related declines in bone mineral density. The association between fracture status and age may reflect declining bone quality with age, a relationship that warrants further investigation using high‐resolution imaging like high resolution peripheral quantitative computed tomography (HR‐pQCT).

Among those who did not undergo ACL‐R, all BML occurrences had resolved in 92.68% of injured people by 1 year, with two lesions in the tibia that did not fully resolve and three newly emerging lesions in the femur. Nevertheless, persistent pain and functional limitations were still reported by some participants, suggesting that BML resolution alone does not capture the full extent of patient recovery. These findings are important because they challenge the assumption that improvements on imaging reflects clinical recovery, highlighting the need to consider factors beyond BML resolution when evaluating patient outcomes.

Younger participants were more likely to undergo ACL‐R in our cohort, which highlights potential age‐related differences in treatment decisions and expectations of function. In examining the KOOS outcomes, there were no significant differences between the surgical and Nonsurgical groups at follow‐up, despite some differences in pain scores. Specifically, while the Nonsurgical group exhibited better pain outcomes (+5.1%, *p* = 0.049), other KOOS subscales, including Symptoms, ADL, Sport, and QoL, did not show meaningful differences between groups. These findings suggest that, on average, the overall benefits of ACL‐R on patient‐reported outcomes may not be substantially superior to those of Nonsurgical management. This aligns with previous research, which has indicated that while surgery may improve certain physical outcomes, it does not necessarily translate to significantly better long‐term quality of life or functional scores. Interestingly, the baseline KOOS scores were strongly predictive of follow‐up outcomes, indicating the value of early functional assessments for predicting future recovery. Specifically, baseline pain levels were highly correlated with follow‐up pain and symptom severity, while baseline sport function demonstrated moderate correlations with both future symptoms and sport function outcomes. These longitudinal associations reinforce the idea that initial levels of pain and sports function are significant predictors of recovery, regardless of whether the individual underwent surgery.

Outside of the acute ACL injury context, BMLs are often present in established OA, where they can arise from subchondral bone insufficiency fractures or abnormal bone remodeling [26, 27]. In established OA, BMLs have been found to strongly correlate with pain [14, 28] and likely result from joint surface stress caused by bone‐on‐bone contact in the absence of adequate cartilage protection, leading to further joint degradation. This mechanism is similar to what may occur in ACL injuries, where abnormal joint mechanics following the rupture could contribute to increased stress in specific regions of the knee, resulting in BML formation. However, the relationship between BML volume and pain may not be as direct in the acute ACL injury setting as it is in chronic ideopathic OA, where BMLs are a hallmark of disease progression.

Several limitations should be considered when interpreting the results of this study. First, it remains unclear whether the associations observed between BML volume and knee pain were confounded by other factors, such as inflammation or concurrent injuries (e.g., meniscal or chondral lesions), which may independently contribute to both BML development and symptom severity. Second, imaging follow‐up was not possible for participants who underwent ACL‐R until long after their surgeries, due to existing surgical related edeama, limiting our ability to assess BML resolution closer to the time of surgical intervention. It is likely that many of these participants experienced lesion resolution before follow‐up imaging, which may have led us to underestimate the rate or timing of BML healing in the surgical group. Third, there were differences in the timing of follow‐up KOOS assessments between groups. Nonsurgical participants were followed for 1 year post‐baseline, while surgical participants completed follow‐up relative to their time of injury (not surgery), which varied between individuals. These differences in follow‐up timing may have introduced bias in comparisons between surgical and nonsurgical groups. Additionally, there are known limitations with the KOOS instrument in ACL populations. Although widely used, KOOS was not designed specifically for acute ligament injuries and includes overlapping content across subscales (Symptoms, Pain, and ADL), which may reduce its construct specificity [29]. These issues complicate the interpretation of subscale scores and may obscure subtle differences in recovery or quality of life that are more relevant in this population.

## 5 Conlusion

This study underscores the complex and multifactorial nature of the relationship between BML volume and knee pain following acute ACL injury. While BMLs were highly prevalent—especially in the LTP and LFC—their volume was not strongly associated with pain, either at baseline or follow‐up. Instead, pain and functional outcomes were more closely predicted by early symptom and sport function scores. Although BML volume was influenced by sex and associated with the presence of depression fractures, it did not predict long‐term outcomes on the KOOS survey. The natural resolution of BMLs in nearly all nonsurgical participants further suggests that BML volume alone is not a primary determinant of persistent pain or dysfunction. Moreover, the frequent co‐occurrence of MCL, LCL, and PCL injuries, along with their associations with age and fracture risk, highlights the interconnected and age‐sensitive nature of traumatic knee injuries. These findings support the need for individualized rehabilitation and treatment strategies that consider the broader biomechanical, inflammatory, and psychological factors influencing recovery. Future work should explore how cartilage damage, inflammation, and bone quality interact with BMLs to better predict long‐term outcomes and guide clinical decisions following ACL injury.

## Author Contributions

The study was conceptualized and designed by Callie E. Stirling, Sarah L. Manske, and Steven K. Boyd. Callie E. Stirling took the lead in data analysis and manuscript preparation. The investigation phase involved contributions from Callie E. Stirling, Nina Pavlovic, and Richard E. A. Walker. Richard E. A. Walker provided MRI reporting and evaluations. Methodological aspects were primarily handled by Callie E. Stirling. Steven K. Boyd secured funding for the study. Resources were managed by Steven K. Boyd. Supervision was provided by Steven K. Boyd. Callie E. Stirling performed visualizations and was responsible for the original draft of the manuscript, with Nina Pavlovic, Sarah L. Manske, Richard E. A. Walker, and Steven K. Boyd providing input. All authors reviewed and approved of the final manuscript.

## Conflicts of Interest

The authors declare no conflicts of interest.

## Data Availability

The data will not be made publicly available to safeguard the confidentiality of participants. All code used for MRI segmentation is publicly available in the following GitHub repository: https://github.com/Bonelab/BML-MRI-segmentation.
